# Trends in depression risk before and during the COVID-19 pandemic

**DOI:** 10.1371/journal.pone.0285282

**Published:** 2023-05-17

**Authors:** Sofia B. Villas-Boas, Justin S. White, Scott Kaplan, Renee Y. Hsia

**Affiliations:** 1 Department of Agricultural and Resource Economics, University of California Berkeley, Berkeley, CA, United States of America; 2 Department of Epidemiology & Biostatistics and the Philip R. Lee Institute for Health Policy Studies, University of California, San Francisco, CA, United States of America; 3 Department of Economics, United States Naval Academy, Annapolis, MD, United States of America; 4 Department of Emergency Medicine and the Philip R. Lee Institute for Health Policy Studies, University of California San Francisco, San Francisco, CA, United States of America; Centre d’Estudis Demogràfics, SPAIN

## Abstract

Using 11 years of the U.S. Centers for Disease Control and Prevention (CDC) Behavioral Risk Factor Surveillance System survey data set for 2011 to 2021, we track the evolution of depression risk for U.S. states and territories before and during the COVID-19 pandemic. We use these data in conjunction with unemployment and COVID case data by state and by year to describe changes in the prevalence of self-reported diagnosis with a depressive disorder over time and especially after the onset of COVID in 2020 and 2021. We further investigate heterogeneous associations of depression risk by demographic characteristics. Regression analyses of these associations adjust for state-specific and period-specific factors using state and year-fixed effects. First, we find that depression risk had been increasing in the US in years preceding the pandemic. Second, we find no significant average changes in depression risk at the onset of COVID in 2020 relative to previous trends, but estimate a 3% increase in average depression risk in 2021. Importantly, we find meaningful variation in terms of changes in depression risk during the pandemic across demographic subgroups.

## 1 Introduction

The COVID-19 pandemic and the accompanying policy responses have had far-reaching health, economic, and social repercussions. The pandemic has exacted an enormous toll on the mental health and well-being of the population. Linking a global pandemic with mental health consequences is prompted by research on past epidemics [1], and there is growing evidence that the COVID-19 pandemic has adversely affected mental health in many countries around the world (see meta-analytic evidence in [2]). A confluence of multiple mechanistic pathways may contribute to worsening mental health during the pandemic: infection or worry about becoming infected; the stress brought about by infection prevention and control measures such as lockdown, self-isolation, and quarantine; the detrimental effects on mental health associated with lost or reduced employment, income, education or social participation; and a lack of access to mental health resources diverted to deal with the global pandemic.

A growing literature has documented the impact of COVID-19 on mental health. Early evidence showed a general worsening in US mental health during the pandemic (see, e.g., [3, 4]). These papers were based on various surveys implemented during the pandemic, such as the Data Foundation’s COVID Impact Survey (CIS) [5] and the Census Bureau’s Household Pulse Survey (HPS) [6, 7]. Using over 40 HPS waves starting in April 2020, [6] found that mental health deteriorated in 2020 and rebounded in 2021 and 2022.

However, the empirical strategies in this literature did not use similar survey instruments for periods before the pandemic. To fully assess how the pandemic affected mental health in the US population on average and across different demographic groups, however, one also requires pre-pandemic data to have a baseline and to account for pre-existing mental health trends. In a related study, [4] followed an alternative approach using a Google trends data set for periods before and after COVID and lockdowns, focusing on search terms relating to mental health in Europe and the US. Comparing well-being-related searches before and after lockdown in 2020 to well-being-related Google searches before and after the same date in 2019, they found increased searches for loneliness, worry, and sadness. Similarly, [15] found the prevalence of depressive symptoms to be higher during COVID than before based on responses for different demographic groups using two waves of the COVID-19 and Life Stressors Impact on Mental Health and Well-being study, conducted in March to April of 2020 and the National Health and Nutrition Examination Survey, conducted from 2017 to 2018. Individuals with fewer social and economic resources and greater exposure to stressors (such as unemployment) reported a more significant burden of depression symptoms. Using recent survey data spanning into 2022, [6] determined that the pandemic’s effects on mental health were concentrated among women, but also found mental health improvements in 2022 for women and college-educated men following vaccine availability.

There is a critical need in the existing literature to investigate mental health during the COVID-19 pandemic in the context of pre–existing trends in depression and other mental health conditions tracked in available data. Our goal in this study is to investigate how the risk of depression has evolved over time, especially during COVID-19. Using 11 years of the US Centers of Disease Control and Prevention’s (CDC) Behavioral Risk Factor Surveillance System (BRFSS) survey data set from 2011 to 2021, together with unemployment data and COVID-19 case data, we follow the evolution of depression for US states before and during the COVID-19 pandemic on average and as unemployment and COVID cases changed by state and year during pandemic years. Given our longitudinal data set spanning over 11 years, we not only investigate the trends during COVID years for different demographic groups, but also assess the long pre-existing trends among different demographic groups. In so doing, we document that mental illness was likely to have become more prevalent even without the onset of the global pandemic, particularly among specific population subgroups. Using depression risk measures by demographic groups, we can analyze the patterns of depression on average as well as for different demographic groups before and during the COVID-19 pandemic. We use these data to discuss evidence of overall changes in depression risk over time and especially during the COVID years of 2020 and 2021, and investigate heterogeneous associations by demographic characteristics.

Our main contributions are twofold: first, we include and assess earlier time periods during the pre-COVID period, and second, we examine trends in depression risk by different subgroups before the onset of COVID and the extent to which these trends change when the COVID-19 pandemic arrived.

## 2 Data and methods

We use the CDC’s public-use BRFSS survey dataset, which is a mail-, telephone-, and cell phone-based survey conducted by state health departments’ in-house interviewers with the technical assistance of the CDC. The BRFSS surveys over 400,000 respondents each year. We use data aggregated to the state level for the period from 2011 through 2021.

The survey is administered by the Division of Behavioral Surveillance in CDC’s Public Health Surveillance and Informatics Program Office; Office of Surveillance, Epidemiology, and Laboratory Services. The target population (aged 18 years and older) for cellular telephone samples consists of people residing in private residence or college housing who have a working cellular telephone in all 50 states and the District of Columbia and three U.S. territories. All responses are self-reported. The BRFSS survey uses a sampling frame of cell phones from the Telecordia database of telephone exchanges. The BRFSS divides the frame of telephone numbers into a set of intervals of telephone numbers, and one 10-digit telephone number is drawn at random. In the sample design, states begin with a single stratum. To provide adequate sample sizes for smaller geographically defined populations of interest, however, many states sample disproportionately from strata corresponding to sub-state regions.

We use a longitudinal data set consisting of a state-level aggregate measure for each question answered. We not only have the overall responses to each topic of interest in the surveillance of risk factors for US citizens’ health (such as diabetes, asthma, and depression), but also the average responses by respondents’ demographics, such as sex, race and ethnicity, age, income, and education levels.

We define risk of depression based on responses to the yes/no question, “Ever told you had a depressive disorder?” (from Questionnaire page 26).

We use two other data sets by state and year. The first is the number of COVID-19 cases obtained from the CDC (Link). We construct a variable of the number of cases per 100,000 people to measure the variation in the severity of the pandemic by state and year. The second data set we use pertains to the state unemployment rate by year. These data come from the Bureau of Labor Statistics (Link). This measure characterizes how unemployment evolves on average by state and year since 2012 and especially during COVID-19 as the economic impact of the pandemic is one predictor of depression risk. Once we combine the depression risk, the unemployment rate, and COVID-19 cases datasets, the data set includes 508 state-year observations from 2012 to 2021 for all US states.

We investigate patterns of the risk of depression for 2020 and 2021 during COVID-19, compared with those in previous years. We model these outcomes using a linear regression model, in which we compare the change in the risk of depression over the different years while controlling for the severity of the pandemic (number of cases per 100,000) and unemployment. Let *D*_*st*_ be the risk of depression in state *s* in year *t* where:

$$\begin{array}{c} D_{st}=\beta_{0}+\beta_{1}UR_{st}+\beta_{2}COVIDCases_{st}+\delta_{t}Year_{t}+\gamma_{s}+\varepsilon_{st} \end{array}$$ (1)

The coefficient *β*_0_ is a constant term for the baseline year of 2018, and measures the average risk of depression in 2018. The variable *UR*_*st*_ measures the unemployment rate in state *s* in year *t*. The variable *COVIDCases*_*st*_ measures the number of COVID-19 cases per 100,000 in state *s* in year *t*. *Year*_*t*_ is a set of indicators equal to one in year *t* for all states, and equal to zero otherwise. Its corresponding coefficient *δ*_*t*_ measures how the risk of depression varies in year *t* relative to base year 2018. The coefficient *γ* is a vector of state fixed effects (i.e., indicator variables). *γ* controls for time-invariant characteristics of state *s* that affect depression risk (e.g., a state’s urbanicity), while each *δ*_*t*_ controls for year-to-year changes in the average risk of depression that are common to all states and are measured relative to the baseline year of 2018 (e.g., the availability of anti-depressant medications). *ε* is an idiosyncratic error comprised of unobserved determinants of changes in depression risk that are not controlled for by the variables specified in the linear model in Eq (1).

The coefficient *β*_1_ measures the marginal effect of unemployment rate on depression risk, and *β*_2_ measures the marginal effect of COVID-cases on depression risk, holding all else constant, that is, controlling for state and time-varying factors that also correlate with changes in depression risk.

Our methodological approach allows us to identify the correlation of unemployment and pandemic severity by state on the change in the risk of depression while explicitly controlling for other confounding factors specific to each state. The share of the local population previously working from home or employed in specific industries are controlled for with *γ*, while year-to-year changes in activity common to all states—motivated by new information on the virus’ spread and nationwide media coverage or federal appeals to social distancing—are captured in the *δ*_*t*_.

In order to test whether there are significant changes in depression risk in 2020 and in 2021 COVID years relative to 2019, the year just before COVID’s onset, we compute the difference between the year coefficients in 2020 and 2021 less the year coefficient in 2019. We test the null that these differences are equal to zero using an F test for a multiple coefficient null hypothesis. Standard errors are clustered by state to account for the correlation in state factors and policies over time.

In addition, we investigate heterogeneity in the pre–COVID and post–COVID depression trend and the correlation of unemployment and the severity of the pandemic by state on the change in the risk of depression among different demographic subgroups using an OLS regression by subgroup as in Eq (1).

## 3 Results

In Table 1, we report summary statistics of depression risk. In the top of Panel A, the overall average risk of depression is 19.3% (n = 508) with a minimum value for a state of 10.7% and the maximum value of 28.8%.

**Table 1:** Summary statistics.

	**Mean**	**Std Dev**	**Min**	**Max**
*Panel A. Depression risk by group*				
Overall	19.3	3.24	10.7	28.8
Female	24.2	4.16	12.4	35.5
Male	14.2	2.53	8	21.7
18–24	20.1	5.52	7.1	36.1
25–34	21.0	4.80	9.5	36.1
35–44	20.0	4.36	9.8	32.7
45–54	20.5	4.05	9.8	31.8
55–64	21.0	3.69	12.7	31.5
65+	14.6	2.48	7.6	20.3
Multiracial, non Hispanic	29.5	7.43	12.4	57
Asian, non Hispanic	9.8	3.65	4.4	24.7
Black, non Hispanic	16.6	3.91	8.2	33
Native American PI, non Hispanic	10.5	2.03	7.7	13.3
Hispanic	18.1	5.76	8.3	41.5
White, non Hispanic	20.5	2.98	13.3	28.4
Less than $15,000	34.8	6.87	17.3	54.6
$15,000-$24,999	25.9	5.28	12.5	40.4
$25,000-$34,999	21.0	4.56	10.8	35.9
$35,000-$49,999	18.5	3.72	7.9	31.4
$50,000+	14.5	2.42	8.3	21.5
Less than Highschool (HS)	24.5	5.85	10.1	41.6
HS or GED	18.9	3.47	8.5	29.6
Some post HS	20.9	3.60	10.5	32.2
College graduate	15.5	2.64	8.9	22.6
*Panel B. Time-varying factors*	**Mean**	**Std Dev**	**Min**	**Max**
Unemployment rate, %	5.30	1.80	2.10	13.80
2019	3.58	0.82	2.11	5.48
2020	7.40	1.90	4.15	13.78
2021	4.88	1.25	2.51	7.37
COVID-19 cases per 100,000	0.73	2.99	0	40.34
2020	0.95	1.06	0	4.98
2021	6.47	7.33	0.25	40.34

Note: Panel A contains summary statistics of depression risk overall and by demographic subgroup. Panel B contains summary statistics for the unemployment rate and COVID-19 cases per 100,000.

We further report in Table 1 summary statistics of depression risk by sex, age, race and ethnicity, household income, and educational attainment. Women had a higher average depression risk (mean 24.2%, n = 508) than men (mean 14.2%, n = 508). Depression risk by age was lowest for the group aged 65 and older (mean 14.6%, n = 508), whereas for the other age groups, depression hovered between 20% and 21%. The multiracial (non-Hispanic) subgroup had the highest average risk of depression by race/ethnicity (mean 29.5%, n = 429), followed by non-Hispanic Whites (mean 20.5%, n = 508). Native Americans and Pacific Islanders (Mean = 10.5%, n = 5) and non-Hispanic Asians (Mean = 9.8%, n = 89) had the lowest risk. Average risk of depression decreased by income, with the highest risk for respondents with income less than $15,000 (mean 34.8%, n = 508) and the lowest risk for those with income above $50,000 (mean 14.5%, n = 458). In terms of education, the highest risk of depression was found for those with less than high school education (mean 24.5%, n = 507), followed by those with some college (mean 20.9%, n = 508), high school or GED (mean 18.9%, n = 508), and the lowest for college graduates (mean 15.5%, n = 508).

In Panel B of Table 1, we report summary statistics for the unemployment rate and the number of COVID-19 cases per 100,000. Over the entire sample, average unemployment was 5.3% with a minimum of 2.1% and a maximum of 13.8%. In 2020 average unemployment of 7.4% was higher than in 2019 (3.6%) with a minimum of 4.2% and a maximum of 13.8%, while average unemployment was again lower in 2021 (4.9%). Average COVID-19 cases were overall 0.7 per 100,000 (minimum of 0 and maximum of 40.3). In 2020, on average, cases were 1.0 per 100,000 in 2020 and 6.5 per 100,000 in 2021.

**Fig 1:**
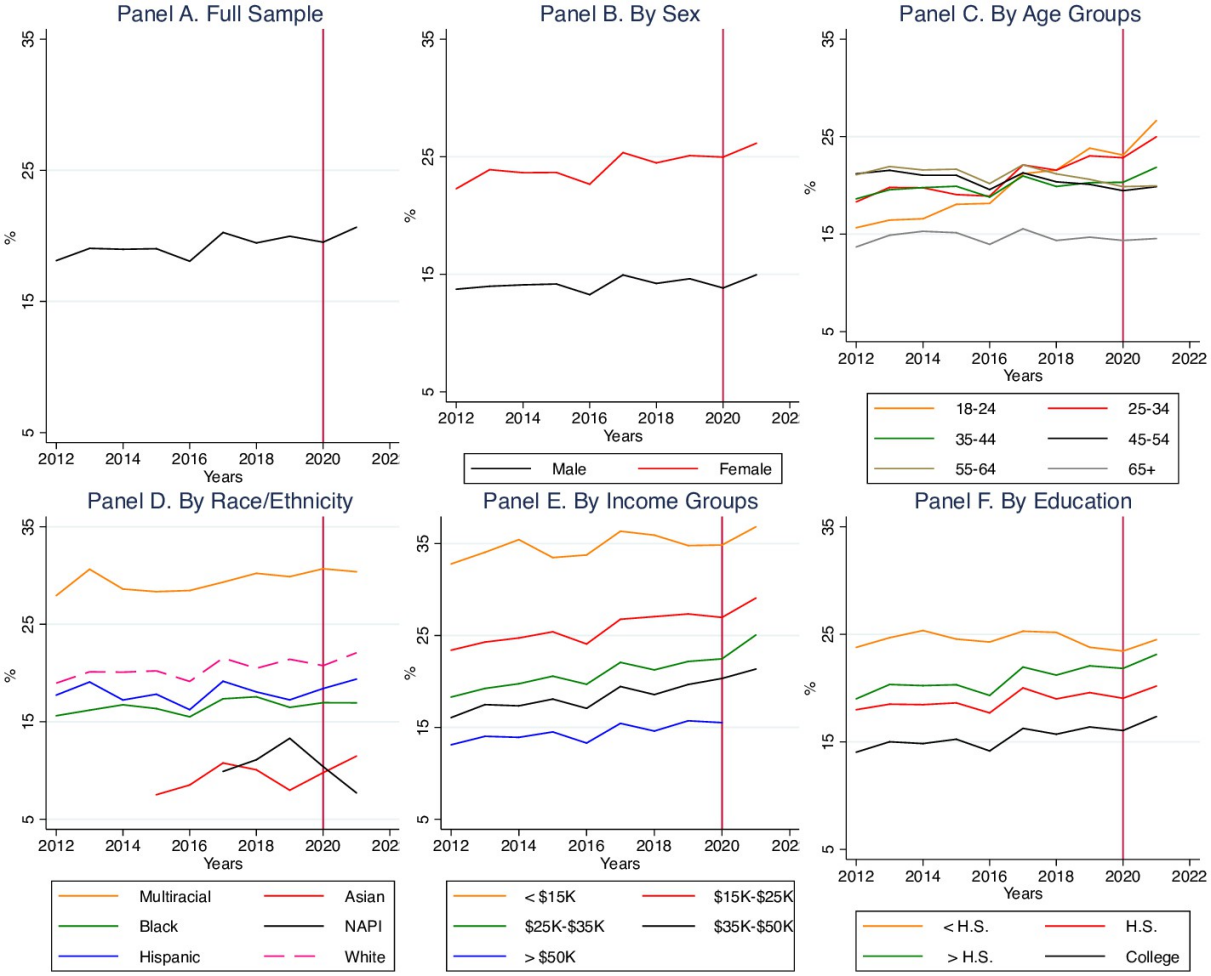
Risk of depression, overall and by demographic subgroups, 2011–2021. Note: BRFSS data, 2011–2021. Panel A is the yearly trend in depression risk. Panel B displays the trends by sex, Panel C by age group, Panel D by racial/ethnic group, Panel E by income group, and Panel F by education group. The vertical red line in each panel indicates the year of the pandemic onset, 2020.

In terms of trends over time in the overall risk of depression depicted in the top left panel of Fig 1 we see that it had an upwards yearly trend. The following panels break up the trends by sex, age, ethnicity, income, and education group. The risk of depression for women was higher than men for all years, and both trended in a parallel fashion. In 2020 male depression risk tipped down and increased again in 2021, while female risk increased always. The younger age groups had a steeper upwards slope in depression risk than the older age groups. Ethnic groups also had a similar yearly prevalence trend except for the NAPI and Asian groups towards the later years, where the risk tipped downward for NAPI and upward for Asians. Finally, the yearly patterns were similar across groups in terms of income and education.

In the full sample, depression risk was trending upward prior to the pandemic from 17.7% in 2011 to 19.9% in 2019. After the pandemic’s onset, depression risk was to 19.5% in 2020 followed by a worsening of depression risk to 20.7% in 2021. Across all demographic subgroups, there was an uptick in the depression risk in 2021 relative to 2020, except for the older age groups and the Native American / Pacific Islander (NAPI) group.

Results for the estimation of Eq (1) for the full sample are given in the column labeled **Overall** in Panel A of Table 2. We see that while depression risk was on average 19.29% in 2018 (the estimate for the constant term), it was already increasing prior to COVID, given the positive and significant estimate in the row labeled “2019.” Specifically, the estimated Coef_2019_ was 0.41 (*p* < 0.1), which means that, on average depression risk in 2019 was 0.41 percentage points (pp) higher than in 2018. We then see that depression risk in 2020 dropped relative to 2019, and it was not significantly different than in 2018 (-0.14 pp, *p* > 0.1). Finally, in 2021, average depression was significantly higher than in 2018 by 1.0 pp (*p* < 0.01). Comparing 2021 to the pre–COVID 2019 depression risk levels, average depression increased significantly by 0.59 pp (Coef_2021_—Coef_2019_ = 1.0 − 0.41 = 0.59, *p* < 0.1). Using the baseline depression risk in 2018 of 19.29%, a 0.59 pp increase translates into an approximately 3% increase in depression incidence. In addition, the variation in unemployment rate ($\hat{\beta_{1}}=0.05,p>0.1)$ and COVID cases ($\hat{\beta_{2}}=0.01,p>0.1)$ were positively but not significantly associated with depression risk.

**Table 2:** Regression analysis of overall evolution of depression risk before and after 2020, adjusted for unemployment rate and COVID cases per 100,000, and subgroup estimates by sex, age (Panel A), ethnicity, income, and education (Panel B).

**Panel A.**	**All**	**Sex**		**Age**						**Race**
		**Male**	**Female**	**18–24**	**25–34**	**35–44**	**45–54**	**55–64**	**65+**	**Mixed**
Un. Rate	0.05	0.10	0.02	-0.14	-0.09	0.10	0.12	0.09	0.16	0.14
	(0.09)	(0.08)	(0.12)	(0.25)	(0.16)	(0.13)	(0.14)	(0.12)	(0.12)	(0.44)
Cases	0.01	0.03+	-0.01	0.02	0.00	-0.01	0.02	0.00	0.00	-0.09
	(0.02)	(0.02)	(0.03)	(0.05)	(0.04)	(0.03)	(0.03)	(0.02)	(0.02)	(0.08)
2019	0.41*	0.35+	0.47	2.12**	1.32**	0.27	-0.37	-0.66*	0.32	-0.24
	(0.21)	(0.20)	(0.29)	(0.62)	(0.49)	(0.41)	(0.42)	(0.29)	(0.25)	(1.39)
2020	-0.14	-0.79**	0.44	2.02*	1.59*	0.08	-1.373+	-1.62*	-0.56	0.01
	(0.38)	(0.36)	(0.52)	(0.85)	(0.66)	(0.57)	(0.69)	(0.61)	(0.52)	(2.20)
2021	1.00***	0.37	1.64***	4.99***	3.46***	1.82***	-0.82+	-1.36***	0.03	0.15
	(0.30)	(0.30)	(0.41)	(0.73)	(0.70)	(0.44)	(0.47)	(0.37)	(0.40)	(1.48)
Constant	19.29***	13.85***	24.43***	22.12***	21.90***	19.54***	19.92***	20.87***	13.77***	29.61***
	(0.41)	(0.37)	(0.51)	(1.13)	(0.73)	(0.51)	(0.58)	(0.52)	(0.48)	(1.95)
Num of Obs. R squared	508	508	508	507	508	508	508	508	508	429
	0.87	0.79	0.86	0.77	0.78	0.81	0.79	0.80	0.73	0.46
**Panel B.**	**Race/ethnicity**				**Income/Education**					**> HS**
	**Asian**	**Black**	**Hisp**	**White**	**< $15K**	**$15–25K**	**$25–35K**	**$35–50K**	**< HS**	
Un. Rate	0.12	0.04	0.06	0.03	0.12	-0.05	0.12	0.08	0.43*	-0.01
	(0.32)	(0.31)	(0.21)	(0.11)	(0.27)	(0.15)	(0.16)	(0.19)	(0.22)	(0.15)
Cases	0.06**	-0.04	0.03	0.04	-0.07	-0.05	0.03	0.03	-0.07	0.07**
	(0.02)	(0.04)	(0.06)	(0.03)	(0.08)	(0.04)	(0.04)	(0.09)	(0.05)	(0.03)
2019	-0.43	-1.03	-0.41	0.84***	-1.14	0.16	0.84*	1.12**	-1.44**	0.75**
	(1.02)	(0.66)	(0.65)	(0.20)	(0.60)	(0.51)	(0.49)	(0.51)	(0.55)	(0.29)
2020	-0.46	-0.72	-0.14	0.14	-1.44	0.16	0.76	1.52*	-3.22***	0.59
	(1.88)	(1.15)	(0.97)	(0.44)	(1.10)	(0.62)	(0.71)	(0.79)	(0.97)	(0.56)
2021	-0.14	-0.45	0.72	1.24***	1.13	2.32***	3.44***	2.49***	-0.88	1.40***
	(0.80)	(0.79)	(0.91)	(0.37)	(1.14)	(0.72)	(0.65)	(0.71)	(0.71)	(0.44)
Constant	9.40***	17.34***	17.77***	20.39***	35.50***	27.27***	20.84***	18.24***	23.60***	21.25***
	(1.32)	(1.38)	(0.94)	(0.47)	(1.14)	(0.65)	(0.74)	(0.84)	(0.85)	(0.65)
Num of Obs. R squared	89	390	459	508	508	508	507	507	507	508
	0.85	0.57	0.71	0.83	0.76	0.79	0.7	0.68	0.75	0.79

Note: Standard errors in parentheses are clustered at the state level.

*p<0.1,

**p<0.05,

***p<0.01.

All regressions include state and year fixed effects. Unemployment rate measures the average unemployment rate by state by year. Cases measures the average by state by year of COVID 19 cases per 100,000. The Constant is the average depression risk in 2018. We do not include estimates for the NAPI racial group due to insufficient observations, as well as for the highest income cohort due to missing data for 2021.

We next turn to estimates of depression risk among demographic subgroups of our sample in the remaining columns of Panels A and B of Table 2. Starting with the sex breakdown in columns with headers **Male** and **Female**, we see that depression risk for women was on average 24.4% in 2018 (the constant estimate) compared with 13.9% for men. For men, depression risk was already increasing prior to COVID, given the positive and significant estimate in the row labeled “2019.” Specifically, the estimated Coef_2019_ was 0.35 (*p* < 0.1), which means that, on average depression risk in 2019 was 0.35 pp higher than in 2018 for men. For women, depression was similar in 2019 to 2018 (0.47 pp, *p* > 0.1). We then see that depression risk in 2020 dropped relative to 2018 for males (−0.79, *p* < 0.05), and it was not significantly different than in 2018 for women (0.44 pp, *p* > 0.1). Finally, in 2021 average depression for women was significantly higher than in 2018 by 1.64 pp (1.64 pp, *p* < 0.01), and not significantly different for men. Comparing 2020 to 2019, depression did not change for women and it decreased for men by 1.1 pp (Coef_2020_− Coef_2019_ = −1.14, *p* < 0.01. Comparing 2021 to the pre-COVID 2019 depression risk levels did not change for males. However, for females, average depression increased significantly by 1.2 pp (Coef_2021_− Coef_2019_ = 1.2, *p* < 0.01). In addition, on average the variation in unemployment rate was not correlated with depression risk for both men and women. However, for men, COVID cases ($\hat{\beta_{2}}=0.03,p<0.1$) were significantly correlated with depression risk.

The estimated coefficients depict interesting patterns in the age sub-samples. First, the two youngest subgroups had the highest 2018 depression risk (the estimated constant is 22.12% for ages 18 to 24 and 21.9% for ages 25 to 34). The 65+ group of respondents had the lowest 2018 risk of depression (the estimated constant is 13.8%). Overall, we saw a significant increase in depression risk even before COVID for the two youngest age groups: in 2019 relative to 2018, depression increased by 2.1 pp for the youngest (18 to 24) cohort and by 1.3 pp for the 25 to 34 year-old cohort. During COVID years, we see that depression risk increased in 2020 relative to 2018 for the age cohorts of 18–24 (2.02 pp, *p* < 0.05) and 25–34 (1.6 pp, *p* < 0.05) year old. This pattern continued in 2021: depression risk in 2021 was higher than in 2018 for the three youngest cohorts (for 18–24: 4.9 pp, *p* < 0.01; 25–35: 3.5 pp, *p* < 0.01; for 35–44: 1.8 pp, *p* < 0.01). After testing for significance in the differences in the 2019 and 2020 coefficients, we conclude that depression risk did not change significantly in 2020 relative to 2019. But, the increase was statistically significant for the three youngest cohorts in 2021 relative to pre–COVID in 2019. Specifically, we estimate that depression risk increased by 2.9 pp (p<0.002) for the 18 to 24 year old cohort, by 2.1 pp for the 25 to 34 year old cohort (p<0.005) and by 1.6 pp for the 35 to 44 year old cohort (p<0.001). These represent clinically and statistically meaningful increases of depression of 13.0%, 9.8%, and 8.0%, respectively, for these age groups in 2021 compared to 2019.

The pre-COVID risk of depression dropped for the 55–64 age cohort by 0.66 pp in 2019 relative to 2018 (−0.66, *p* < 0.1). For this group, as well as for the other two oldest cohorts, depression risk dropped in 2020 relative to 2018 (for 45–54: −1.37 pp, *p* < 0.1; for 55–64: −1.62 pp, *p* < 0.05; for 65+: −0.56 pp, *p* > 0.1). This pattern continued in 2021, for the 45–54 and the 55–64 cohorts depression risk in 2021 was lower than in 2018. Comparing 2020 and 2021 to 2019, we conclude first that depression did not change significantly in 2020 nor in 2021 relative to 2019 for the 45 to 54 year old cohort. Second, for the 55 to 64 year old cohort depression risk dropped by 1.0 pp in 2020 (p<0.1) and 0.7 pp in 2021 (p<0.03) relative to 2019. Finally, for the oldest age group we see depression risk dropping in 2020 relative to 2019 by 0.8 pp (p<0.05) but not in 2021 relative to 2019. To conclude the age cohort analysis, we find that, on average the variation in unemployment rate and COVID cases are not correlated with depression risk for any of the age subgroups.

Turning now to race and ethnicity in the last column of Panel A and in Panel B of Table 2, we see that the Mixed Race followed by the White cohort had the highest baseline depression risk. In contrast, the Asian group had the lowest (constant estimates of 20.4% and 9.4%, respectively). During the 2019, 2020, 2021 periods, depression did not change significantly relative to 2018 for Mixed race, Asian, Black, and Hispanic subgroups. We only estimate significant year fixed effects for the White subgroup. Even before COVID, we estimate that depression risk in 2019 was higher than in 2018 for the White subgroup (0.84 pp, *p* < 0.01). However, when comparing the change in 2020 relative to 2019 and the change in 2021 relative to 2019, none of the changes in depression risk was significant for White respondents between post-COVID periods and 2019. As a caveat, we do not have enough observations to be able to estimate the model (with all the fixed effects) for the NAPI cohort.

To conclude the racial/ethnic cohort analysis, while we estimated no association between depression and unemployment for any ethnic groups, for Asians, more COVID cases were positively correlated with depression risk (${\hat{\beta }}_{1}=0.056,p<0.05$).

In terms of income, we estimated a higher average baseline depression risk for lower-income cohorts (constant estimate of 35.5% for less than $15K and constant estimate of 27.3% for $15K-$25K) than for higher-income cohorts (constant estimate of 18.2% for $35–50K). Even before COVID, in 2019 depression risk worsened relative to 2018 for all income groups and significantly for the higher income cohorts. The estimated coefficient for 2019 was equal to 0.84 pp (p<0.1) for $25–35K, and 1.1 pp (p<0.05) for the income cohort of $35–50K. We estimate there to be no change in 2020 relative to 2018 for all income cohorts except the highest ones—the risk of depression in 2020 relative to 2018 was 1.5 pp higher for the $35–50K (and also for the larger than $50K cohort whose column is omitted due to lack of 2021 data). In 2021 relative to 2018 all income cohorts with available data experienced a significant increase in depression risk, as all coefficients are positive and significant in the row “2021.”

However, when comparing the change in 2020 relative to 2019, and subtracting the 2019 coefficient from the 2020 one, we estimate that there was no significant change in depression risk for any income group. In terms of the change in 2021 relative to 2019, we subtract the 2019 coefficient from the 2021 coefficient. After testing for the significance in these differences in coefficients, we conclude first that the risk increased in 2021 relative to 2019 by 2.2 pp (p<0.003), reflecting a relative increase of 8.0% for the $15–25K cohort. In 2021, risk increased by 2.6 pp for the $25–35K cohort relative to 2019 (p<0.000), a relative increase of 12.5%. It also increased by 1.2 pp for the $35–50K cohort but not significantly (p<0.12). To conclude the income cohort analysis, we found no association between depression risk and unemployment and COVID pandemic severity for each income group.

Finally, examining the last columns of Panel B of Table 1 by education level (<HS vs. >HS) at baseline in 2018, respondents with more than high school had a lower depression risk (21.3%) than respondents who had less than high school education (23.6%). Depression risk moved in opposite directions before COVID for both cohorts. It dropped in 2019 relative to 2018 for those with less than high school (-1.4 pp, *p* < 0.05), while it increased for those with more than high school education (0.75 pp, *p* < 0.05). Depression risk was lower in 2020 than in 2018 for those with less than high school (-3.2 pp, *p* < 0.01) and not significantly different in 2020 relative to 2018 for the more than high school education cohort (*p* > 0.1). While 2021 had no significant change for the less than high school cohort, for those with more than high school depression risk increased in 2021 relative to 2018 by 1.4 pp (*p* < 0.01).

When comparing the change in 2020 relative to 2019, and subtracting the 2019 coefficient from the 2020 one, we estimate that there was a significant decrease in depression risk for the less than high school group (p<0.07) and no significant change for the group with more than high school. In terms of the change in 2021 relative to 2019, we subtract the 2019 coefficient from the 2021 coefficient. After testing for the significance in these differences in coefficients, we conclude first that the risk did not change significantly for the less than high school cohort. In 2021 it increased by 0.7 pp (p<0.1) for the cohort with more than high school education. To conclude the education cohort analysis, we find a positive correlation between depression and unemployment for the cohort with less than high school, and a positive correlation between depression and COVID case severity for the cohort with more than high school education.

The overall results and subgroup heterogeneous associations were robust to a specification that used the natural logarithm of the dependent variable as reported in S1 Appendix Table 3.

## 4 Discussion and conclusion

Using a dataset by state and year on a measure of depression risk from the BRFSS from 2011 to 2021 to employ an empirical approach to estimate how the pandemic, its severity, and unemployment correlated with the evolution of depression risk in the United States, we found no overall significant changes in depression risk during the first year of COVID-19 in 2020, having conditioned on unemployment, COVID cases (pandemic severity) by state and year, and state and time fixed effects. However, we did find an overall 3% increase (0.59 pp increase on a base of 19.29%) in average depression risk in 2021 relative to pre-COVID 2019 depression levels. Overall, the average risk of depression was 19.3%, with the prevalence of reported depression higher in females, younger, non-Hispanic multiracial, low-income, and less educated cohorts. In addition, we found heterogeneity in the estimated effects of the COVID pandemic by demographics. Depression risk decreased for men in 2020 and it increased significantly for women, younger cohorts, and lower income respondents during COVID and especially in 2021. While unemployment was not associated with overall depression risk, we estimated that the severity of the pandemic, measured by COVID cases per 100,000, was positively correlated with depression for men.

In terms of the COVID-19 pandemic among underrepresented minorities, we found no COVID-related changes in mental health trends for these minority cohorts. However, depression worsened during COVID for most income cohorts. Finally, education cohorts exhibited counter-intuitive mental health trends even before COVID, and those trends continued post COVID; namely, that there was a significant decline in depression risk for those with less than high school education, while the trend was positive for those with more than high school education. However, unemployment during this time period was associated with a higher risk of depression for those with less than a high school education, and not for those with more than a high school education.

We find that depression risk had been increasing in the US in years preceding the pandemic. In 2019, before COVID, we estimate that the risk of depression increased by 2.2% relative to 2011, which is consistent with other studies [6, 8, 9]. Moreover, this increase held for many population demographic subgroups with the exception of the oldest age cohort that had a less steep positive trend in depression risk than the younger age cohorts. These patterns are consistent with evidence from [9] that youth mental health was worsening even before the COVID-19 pandemic and that mental illness was also rising among adults.

While we find that the pandemic years were significantly associated with increases in overall depression risk in 2021, the 2020–2021 COVID years affected various demographic subgroups of the US population differently. Specifically, the largest association of the pandemic on increased depression risk was in 2021 relative to 2019 for 18 to 24 year-old cohorts and those with incomes $15-$25K who, respectively, experienced a 13% and 8% increase in depression risk.

These results are consistent with evidence from the HPS survey in [6, 10]. In particular, [10] tracked the change in anxiety and depressive disorders and taking prescription medications or getting counseling between August 2020 and February 2021. They noted that those under age 30 experienced an increase in the percentage reporting depressive symptoms on the majority of days. We believe that we present novel income findings as we did not find any contemporaneous studies by income to which we could compare our findings, although associations between depression and low income have been previously documented [11].

In terms of the hypothesis that the pandemic disproportionately hurt minority racial/ethnic groups more severely, we did not find this to be the case when controlling for unemployment, COVID case rates, and state and time fixed effects. The baseline depression risk for Whites was higher than for minority groups. While depression risk was lower for Black individuals and higher for White individuals in 2019 relative to 2018, we did not estimate changes for any minority groups during COVID, and the increase for the White cohort during COVID was not significant. Our findings are consistent with [12], which estimates no mental health disparities during the early months of the pandemic. Other studies report differences among Hispanics, Blacks, and Whites, but without statistical significance [13–15].

The estimated heterogeneous associations for specific demographic subgroups are revealing. For instance, the younger age sub-samples experienced an increase in depression risk, while older age groups did not. This is potentially consistent with two mechanisms: on the one hand, these were the individuals most likely to be enrolled in school or working in front-line service jobs, or with young children, faced with continuing their schooling, work, and care-giving in the wake of the pandemic. On the other hand, this was also the age group entering the job market or working in states that stopped hiring due to the pandemic-induced economic shutdown. The results for increased depression risk among women are also consistent with evidence that women were harder hit due to leaving the labor force and also having added care duties during the 2020–2021 COVID years (see e.g., [6]). Finally, we find increased depression for low income cohorts as well as for higher income cohorts.

This paper contributes to the existing literature along several fronts: first, by providing new empirical evidence on the evolution of depression during COVID. We used data on depression risk by year and state with unemployment data and COVID case severity data. We use a longitudinal fixed-effects control structure, and also control for state-by-year varying unemployment as well as COVID case rates that could differ over time and by state. In a related paper, [16] used three waves of a survey implemented in March, April, and May 2020 and the state-level variation of stay-at-home mandate implementation to estimate a significant negative effect of lock-downs on mental health outcomes. We investigated post-COVID mental health patterns as well but also evaluated pre-existing trends. Our second contribution was to perform the analysis for key demographic subgroups, using their own baseline trends.

Using data over 10 years, we not only investigate trends in depression incidence during the COVID years for different demographic groups, but also assess the long pre-existing trends in depression incidence among different demographic groups. In so doing, we document that mental illness was increasing well before the onset of COVID–since 2011–and there was a sharp increase in 2017. Interestingly, we also found that reported depression risk did not increase immediately at the beginning of the COVID pandemic. Rather, compared to 2019, depression risk increased only in 2021, not in 2020, which marked the start of the pandemic. Future work may look to investigate the reason for this lag.

Future research could also investigate reasons explaining our findings that not all subgroups had significant increases in depression risk associated with unemployment changes and with the severity of the pandemic, as measured by the number of COVID cases by state and time.

Our paper has several limitations. First, our measure of depression risk is based on an affirmative answer to the question of whether a respondent was told they had a depressive disorder. This is a likely to under-state the actual prevalence of depression disorders, in general, and especially during COVID when medical resources were diverted to dealing with a global pandemic crisis. Further, respondents might have been less likely to seek mental health care during the pandemic when many people avoided medical settings to reduce the risk of contracting COVID-19. Second, we did not have monthly data by state on mental health risk, which would allow us to control for and investigate seasonal aspects of mental health and take advantage of the timing of adoption of stay-at-home and other mitigation policies by states during different months in 2020 in the empirical strategy. Access to monthly data would also allow us to investigate the mental health effects of the availability and the implementation of vaccines, as in [6]. Future research could use person-level data and investigate the persistence of associations using additional years of data.

In conclusion, we found a general increase over time in the risk of depression in the US population over the 10-year period from 2011–2021, which was accentuated during the COVID pandemic in 2021 even after controlling for other factors. In terms of heterogeneity, we did find clinically and statistically meaningful increases in depression risk during the COVID pandemic for the younger age groups of 18–24, 25–34, and 35–44, as well as increased depression in individuals with more than a high school education. While women depression increased significantly in 2021 relative to pre–pandemic trends, men experienced decreases in depression risk in 2020 relative to 2019 but worsened risk in 2021, resulting in similar levels than in 2019 in terms of depression risk for men.

## Supporting information

S1 Appendix (PDF)

## References

[1] Shiltz JM, Baingana F and Neria Y. The 2014 Ebola outbreak and mental health: current status and recommended response. AMA. 2015 Feb 10;313(6):567–8. doi: 10.1001/jama.2014.17934 25532102

[2] World Health Organization. Mental Health and COVID-19: Early evidence of the pandemic’s impact. 2022. https://www.who.int/publications/i/item/WHO-2019-nCoV-Sci_Brief-Mental_health-2022.1.

[3] Holingue C, Kalb LK, Riehm KE, Bennett D, Kapteyn A, Veldhuis C, et al. Mental distress in the United States at the beginning of the COVID-19 pandemic. American Journal of Public Health. 2020;110(11 ):1628–34. doi: 10.2105/AJPH.2020.305857 32941066 PMC7542294

[4] Brodeur A, Clark A, Fleche S, and Powdthavee N. Lockdowns and well-being: Evidence from Google trends. Journal of Public Economics. 2021 Dec;193:104346. 33281237 10.1016/j.jpubeco.2020.104346 PMC7703221

[5] Wozniak, A, Willey, J, Benz, J and Hart, N. COVID Impact Survey: Version 1. 2020. Chicago, IL: National Opinion Research Center.

[6] Blanchflower DG and Bryson A. Covid and mental health in America. PLoS ONE. 2022; 17(7): e0269855.1970. 35867704 10.1371/journal.pone.0269855 PMC9307159

[7] US Census Bureau. Household Pulse Survey: measuring social and economic impacts during the COVID-19 pandemic. 2020. https://www.census.gov/householdpulsedata.

[8] Swaziek Z and Abigail Wozniak A. Disparities Old and New in US Mental Health during the COVID-19 Pandemic. Fiscal Studies. Special Issue: The COVID-19 Economic Crisis. 2020;41(3):709–732. 10.1111/1475-5890.12244 PMC7753757 33362315

[9] Mental Health America COVID-19 and Mental Health: A Growing Crisis. 2021.

[10] Vahratian A, Blumberg SJ, Terlizzi EP, Schiller J.S. Symptoms of anxiety or depressive disorder and use of mental health care among adults during the COVID-19 Pandemic—United States, August 2020–February 2021. MMWR Morbidity Mortality Weekly Report. 2021. 70:490–494. doi: 10.15585/mmwr.mm7013e2 33793459 PMC8022876

[11] Zimmerman F., Katon W. Socioeconomic status, depression disparities, and financial strain: what lies behind the income-depression relationship?. Health Economics. 2005. 14 (12): 1197–1215. doi: 10.1002/hec.1011 15945040

[12] Goldmann E, Hagen D, El Khoury E, Owens M, Misra S, Thrul J. An examination of racial and ethnic disparities in mental health during the Covid-19 pandemic in the US South. J Affect Disord. 2021;295:471–478. doi: 10.1016/j.jad.2021.08.047 34507228 PMC8424176

[13] Breslau J., Finucane M.L., Locker A.R., Baird M.D., Roth E.A., Collins R.L. A longitudinal study of psychological distress in the United States before and during the COVID-19 pandemic. Prev. Med. 2021;143. doi: 10.1016/j.ypmed.2020.106362 33388325 PMC9753596

[14] Ettman C.K., Abdalla S.M., Cohen G.H., Sampson L., Vivier P.M., Galea S. Prevalence of depression symptoms in US adults before and during the COVID-19 pandemic. JAMA Netw. Open. 2020;3. doi: 10.1001/jamanetworkopen.2020.19686 32876685 PMC7489837

[15] McKnight-Eily L.R., Okoro C.A., Strine T.W., Verlenden J., Hollis N.D., Njai R. et al. Racial and ethnic disparities in the prevalence of stress and worry, mental health conditions, and increased substance use among adults during the COVID-19 Pandemic—United States, April and May 2020. MMWR Morb. Mortal. Wkly. Rep. 2021;70:162–166. doi: 10.15585/mmwr.mm7005a3 33539336 PMC7861483

[16] Adams-Prassl A, Boneva T, Golin M, and Rauh C. The impact of the coronavirus lockdown on mental health: evidence from the United States. Economic Policy. 2022. eiac002.

