# Supplementary material for: Trends in depression risk before and during the COVID-19 pandemic
Source: PLoS One. 2023 May 17;18(5):e0285282. doi: 10.1371/journal.pone.0285282 (PMC10191294; doi:10.1371/journal.pone.0285282)
Supplement: S1 Appendix — (PDF) [file pone.0285282.s001.pdf]

Supporting information

S1 Table. Table 3 reports the estimates for the specification in logs. The results in logs are consistent with the findings in levels from Table 1.

Interestingly, holding all else equal, we estimate that unemployment rate was positively correlated with depression risk for respondents with less than high school (0.43, p<0.10) and we find no significant correlations for other demographic subgroups. In terms of COVID cases, we estimate a positive correlation between cases and depression risk for men (0.03, p<0.10), for respondents with more than high school (0.07, p<0.05), and for Asian respondents (0.06, p<0.05).

The results using the natural logarithm of depression risk also allowed us to interpret the associations in terms of percent changes in depression risk straightforwardly, as we report in Appendix Table 3. To summarize, we estimate that depression was already increasing in 2019 relative to 2018 by 2.2% ($p < 0.05$) in the **Overall** column. Then we find that, on average, depression risk dropped in 2020 relative to 2019, but not significantly, given that the difference between the percent change in 2020 and the percent change in 2019 relative to 2018 (-0.011-0.022) had a p>0.17. Ultimately we estimate an **Overall** 2% increase in average depression in 2021 relative to 2019 (computed as the difference between the "2021" coefficient of 0.044 and the "2019" coefficient of 0.022).

In terms of magnitude, depression risk dropped significantly by 7% for men in 2020 relative to 2019 but was 2.7% lower in 2021 relative to 2019. For women, we estimate no

Table 3. Robustness of Results Regression in Logs - analysis of overall evolution of the natural Log of depression risk, and heterogeneity by sex, age (Panel A), ethnicity, income, and education (Panel B), controlling for Unemployment and COVID cases by state and year

**Panel A.**	**Overall**	**Male**	**Female**	**18-24**	**25-34**	**35-44**	**45-54**	**55-64**	**65+**
Unempl. Rate	0.004	0.008	0.003	-0.004	-0.004	0.008	0.005	0.003	0.010
	(0.005)	(0.007)	(0.005)	(0.012)	(0.008)	(0.007)	(0.007)	(0.006)	(0.009)
COVID Cases	0.001	0.003***	0.000	0.005***	0.003*	0.001	0.001	-0.000	0.000
	(0.001)	(0.001)	(0.001)	(0.002)	(0.002)	(0.002)	(0.002)	(0.001)	(0.002)
2019	0.022**	0.027*	0.020*	0.097***	0.061***	0.014	-0.024	-0.028**	0.025
	(0.011)	(0.016)	(0.011)	(0.032)	(0.021)	(0.021)	(0.023)	(0.014)	(0.017)
2020	-0.011	-0.057**	0.012	0.083*	0.079**	-0.007	-0.071**	-0.073**	-0.035
	(0.019)	(0.028)	(0.021)	(0.042)	(0.032)	(0.031)	(0.033)	(0.030)	(0.036)
2021	0.044***	0.021	0.058***	0.183***	0.134***	0.079***	-0.043	-0.060***	0.004
	(0.016)	(0.021)	(0.017)	(0.034)	(0.030)	(0.021)	(0.026)	(0.018)	(0.027)
Constant	2.938***	2.607***	3.172***	3.062***	3.060***	2.936***	2.975***	3.022***	2.607***
	(0.023)	(0.030)	(0.022)	(0.059)	(0.039)	(0.029)	(0.032)	(0.026)	(0.036)
Num of Obs.	508	508	508	507	508	508	508	508	508
R squared	0.868	0.783	0.870	0.772	0.786	0.804	0.788	0.797	0.716
**Panel B.**	**Asian**	**Black**	**Hisp**	**White**	**$15-25K**	**$25-35K**	**$35-50K**	**< HS**	**> HS**
Unempl. Rate	0.014	0.001	0.005	0.002	-0.001	0.009	0.005	0.019*	0.001
	(0.036)	(0.018)	(0.013)	(0.006)	(0.007)	(0.008)	(0.011)	(0.010)	(0.007)
COVID Cases	0.007**	-0.002	0.003	0.003*	-0.000	0.004*	0.002	-0.003	0.004***
	(0.002)	(0.002)	(0.003)	(0.001)	(0.001)	(0.002)	(0.005)	(0.003)	(0.001)
2019	-0.069	-0.074*	-0.014	0.041***	0.007	0.039	0.056**	-0.058**	0.035***
	(0.126)	(0.040)	(0.037)	(0.010)	(0.019)	(0.024)	(0.027)	(0.023)	(0.013)
2020	-0.058	-0.032	-0.013	0.005	-0.001	0.021	0.072*	-0.141***	0.022
	(0.191)	(0.065)	(0.052)	(0.022)	(0.024)	(0.036)	(0.041)	(0.044)	(0.026)
2021	-0.005	-0.030	0.030	0.057***	0.067**	0.124***	0.117***	-0.043	0.055***
	(0.083)	(0.044)	(0.042)	(0.018)	(0.027)	(0.026)	(0.036)	(0.031)	(0.018)
Constant	2.180***	2.836***	2.816***	2.999***	3.281***	3.002***	2.885***	3.121***	3.039***
	(0.150)	(0.081)	(0.062)	(0.024)	(0.026)	(0.036)	(0.047)	(0.040)	(0.032)
Num of Obs.	89	390	459	508	508	507	507	507	508
R squared	0.766	0.586	0.703	0.823	0.802	0.697	0.691	0.740	0.791

Standard errors in parentheses are clustered at the state level. * p<0.10 ** p<0.05 *** p<0.01. All regressions have state and year-fixed effects.

association in 2020 relative to 2019, but depression increased on average for women by 3.8% in 2021 relative to 2019. Specifications in logs have also the same estimated associations among age cohorts. Younger cohorts had the highest percent increases in depression risk. For instance, the biggest percent increase in 2021 relative to 2019 was for the 18 to 24-year-old cohort. They experienced an 8.6% increase (the difference between the 2021 and the 2019 coefficients in the log specification). Once again, in this log specification, we find no differences among racial/ethnic minority groups, all income groups have depression increases in 2021 relative to 2019, and depression worsens during COVID years for cohorts with more than high school. It improves for those with less than high school. The second largest association in the risk of depression was an increase in 2021 relative to 2019 for those with incomes $15-$25K who experienced a 6.7% increase in the risk of depression (the estimate of the 2021 year fixed effect in the log specification in Table 3).
